# Acute Kidney Injury Requiring Dialysis in McArdle Disease: A Case Report of a Rarely Seen Presentation

**DOI:** 10.7759/cureus.22055

**Published:** 2022-02-09

**Authors:** Hugo Veiga, Rita Gouveia, Beatriz Xavier, Patrícia Lourenço

**Affiliations:** 1 Internal Medicine, Centro Hospitalar Universitário de São João, Porto, PRT; 2 Internal Medicine, Faculdade de Medicina da Universidade do Porto, Porto, PRT

**Keywords:** agomelatine, hemodialysis, acute kidney injury, rhabdomyolysis, mcardle disease

## Abstract

McArdle disease is a genetic disorder that leads to impaired glycogenolysis in the muscle, resulting in exercise intolerance, fatigue, myalgias, and basal elevation of creatine kinase (CK). We report a case of a young woman with McArdle disease who had an episode of acute kidney injury (AKI) requiring temporary hemodialysis (HD), with subsequent complete recovery of renal function. We aim to report a rare clinical presentation of an already rare disease and discuss the possible causes involved; therefore, contributing to a better knowledge of the disease.

## Introduction

McArdle disease or glycogen storage disease type V is an autosomal recessive genetic condition that results from mutations in both alleles of the PYGM gene. This leads to a deficiency of myophosphorylase, an essential enzyme for glycogenolysis in the muscle. When this condition and the consequent enzyme deficiency are present, the muscle is unable to break down and use glycogen, resulting in its accumulation within the cells. This abnormal accumulation causes the typical symptoms of the disease like exercise intolerance, fatigue, and myalgias. Virtually all patients have resting elevations in creatine kinase (CK) [1,2] and frequent episodes of rhabdomyolysis with myoglobinuria, sometimes complicated with acute kidney injury (AKI). According to the Spanish national registry, AKI occurs in 4% of cases [1], and in a review of patients in the United Kingdom, the reported prevalence was 11% [2]. Despite being relatively frequent, most AKI cases are mild and very rarely require hemodialysis (HD). In fact, after an extensive literature review, only eight cases of rhabdomyolysis-induced AKI requiring HD have been described in individuals with McArdle disease in the past two decades [3].

We report a case of a young woman with McArdle disease who had an episode of AKI requiring temporary HD, with subsequent recovery of renal function. We aim not only to report a rare clinical presentation of an already rare disease but also to discuss the possible causes involved; therefore, contributing to a better knowledge of the disease.

## Case presentation

A 21-year-old young woman, diagnosed with McArdle disease at age six, presented excellent quality of life despite the decreased tolerance to physical activity and fatigue. She had frequent past episodes of myalgia and rhabdomyolysis, sometimes requiring intravenous fluids on an outpatient basis; the usual triggers for such episodes were more intense physical activity or trauma. Regardless of these episodes of rhabdomyolysis, her renal function was never impaired. She also presented a medical history of multiple food allergies, rhinitis to mites and pollen, bronchial hyperreactivity treated with formoterol + budesonide as and when required, and a depressive syndrome treated with fluoxetine 40 mg/day. Her assistant psychiatrist had recently (the previous week) started clonazepam 0.5 mg/day and agomelatine 25 mg/day. She was on oral contraception with ethinylestradiol + gestodene for a long time.

In May 2021, she went to the emergency department (ED) after five episodes of loss of consciousness, preceded by nausea, dizziness, and palpitations. In one of the episodes, she had clonic movements of the lower limbs, but no other movements, no loss of sphincter continence, or confusion after the episode were noted. She additionally complained of myalgias for some weeks, with no trauma or physical effort associated. She had a documented episode of rhabdomyolysis in the previous month, with the need for intravenous fluids; she had fully recovered. In the ED, she presented with blood pressure (BP) of 98/54 mmHg, heart rate (HR) of 70 bpm, peripheral O2 saturation of 99%, and was apyretic at 36.5ºC. Laboratory tests showed plasmatic creatinine of 1.76 mg/dL, CK > 40,000 U/L, myoglobin > 4,144 ng/mL, aspartate aminotransferase (AST) of 1,216 UI/L, alanine aminotransferase (ALT) of 288 U/L, and lactate dehydrogenase (LDH) of 5,343 U/L. The other parameters including blood counts, ionogram, inflammatory markers, bilirubin, coagulation tests, and thyroid function were within the normal range (as can be seen in Table 1). The urinalysis showed hemoglobinuria, without leukocyturia or nitrituria. Severe acute respiratory syndrome coronavirus 2 (SARS-CoV-2) screening was negative. The electrocardiogram (EKG) showed a normal sinus rhythm. A cranioencephalic computed tomography (CE-CT) scan was performed and showed no alterations. Renal ultrasound was also normal. Intensive intravenous fluid therapy was started and all the recent medications were suspended; only the usual 40 mg/day fluoxetine was maintained.

**Table 1:** Laboratory findings on admission.

Laboratory variables	Measured values	Reference interval
Hemoglobin (g/dL)	17.3	12.0-15.0
Leukocytes (/µL)	11,540	4,500-11,000
Platelets (/µL)	144,000	150,000-400,000
Sodium (mmol/L)	137	136-146
Potassium (mmol/L)	4.8	3.5-5.1
Chloride (mmol/L)	102	101-109
Calcium (mEq/L)	3.8	4.4-5.3
Phosphorus (mg/dL)	6.1	2.5-4.5
Magnesium (mg/dL)	2.6	1.9-2.5
Urea (mg/dL)	45	10-50
Creatinine (mg/dL)	1.76	0.66–1.09
Glucose (mg/dL)	116	76-110
Total bilirubin (mg/dL)	0.6	0.3-1.2
Aspartate aminotransferase (IU/L)	1,216	10-31
Alanine aminotransferase (IU/L)	288	10-31
Gamma-glutamyltransferase (IU/L)	9	7-32
Alkaline phosphatase (IU/L)	55	30-120
Lactate dehydrogenase (IU/L)	5,343	135-225
C-reactive protein (mg/L)	17.5	<15.0
Creatine kinase	>40,000	10-149
Myoglobin	>4,144	14.3-65.8

In the following hours, she evolved with anuria and metabolic acidosis (pH: 7.31; partial pressure of carbon dioxide: 32 mmHg; bicarbonate: 16 mmol/L; lactate: 0.5 mmol/L), and a strategy of aggressive diuretic stimulation was initiated. She presented no response, remained anuric, and her plasma creatinine levels rose from 1.76 mg/dL to 4.73 mg/dL and 5.71 mg/dL in 24 and 48 hours, respectively. At this point, CK was 93,692 UI/L, myoglobin was >4,144 ng/mL, AST was 818 UI/L, ALT was 253 UI/L, and LDH was 2,163 UI/L. She was severely hypervolemic. A temporary central venous catheter (CVC) was placed in the right femoral artery and HD was initiated. Screening for other pathologies was carried out. Antinuclear antibody (ANA), anti-double-stranded deoxyribonucleic acid (anti-dsDNA), anti-extractable nuclear antigen, antineutrophil cytoplasmic antibody (ANCA), and anti-glomerular basement membrane (anti-GBM) antibodies were negative. She presented no complement factors consumption and the serum protein electrophoresis was normal. The patient remained anuric for five days. She recovered diuresis on the sixth day, with increasing volumes of urine output since then. HD was suspended after a total of five sessions. Creatinine levels continued to rise in the following two days (to a maximum of 8.93 mg/dL), but with increasing urine outputs. After that, a sustained decrease in creatinine, CK, and myoglobin was observed and she was discharged two weeks after admission in the ED, asymptomatic, with a plasma creatinine of 2.78 mg/dL, AST of 18 U/L, ALT of 34 U/L, CK of 470 U/L, and myoglobin of 73 ng/mL, with normal blood counts and a normal ionogram. On reassessment two weeks after discharge, she remained asymptomatic and her plasma creatinine was 0.9 mg/dL.

## Discussion

We present a case of AKI induced by myoglobinuria in a patient with a predisposition to episodes of rhabdomyolysis due to McArdle disease. In our patient, despite the history of previous episodes of rhabdomyolysis, this was the first time renal function was seriously impaired and HD was necessary. The trauma resulting from the falls seems to have been the main trigger of muscle damage. The muscle injury was, however, greater than usual, with greater myoglobinuria and severe kidney injury with anuria. We, therefore, hypothesized that the recently started agomelatine may have played a role in the kidney injury. Agomelatine is an antidepressant that acts as a potent agonist of melatonin receptors (MT1 and MT2), but also as an antagonist of serotonin receptors (5-HT2C), having hepatic metabolism and 80% renal excretion, mainly of its metabolites [4,5]. Although no adverse effects related to kidney or muscle damage have been described in the case of agomelatine [5], it is possible that in patients with McArdle disease or even other muscle diseases, there is an increased susceptibility to the effect of this drug, acting synergistically with trauma to trigger more severe rhabdomyolysis. Additionally, adverse muscular effects have been described with agomelatine use [6], although such effects are not included in the summary of the drug’s characteristics [5]. Also, the increase in liver transaminases is a common effect of agomelatine, suggesting that the newly introduced drug was having some effect, at least with regard to liver toxicity. Moreover, it is worth mentioning that one of the few studies that reported cases of AKI requiring HD in patients with McArdle disease corresponds to patients with rhabdomyolysis associated with an antidepressant, although the drug involved was venlafaxine [3].

## Conclusions

We believe this case contributes to a greater knowledge of the disease and confirms the reversible and benign nature of AKI described in these cases. The time until renal function recovery (in the case of our patient required five HD sessions) is also in line with that described in the literature (three to nine HD sessions). Therefore, we share an expected outcome of a rare manifestation of an also rare metabolic disease - the McArdle disease.

## References

[1] Lucia A, Ruiz JR, Santalla A (2012). Genotypic and phenotypic features of McArdle disease: insights from the Spanish national registry. J Neurol Neurosurg Psychiatry.

[2] Quinlivan R, Buckley J, James M (2010). McArdle disease: a clinical review. J Neurol Neurosurg Psychiatry.

[3] Torner S, Tinel C, Soltani Z, Rifle G, Mousson C (2016). Rhabdomyolysis with acute renal failure requiring dialysis in McArdle disease: a role for the antidepressant venlafaxine?. J Clin Psychopharmacol.

[4] Green B (2011). Focus on agomelatine. Curr Med Res Opin.

[5] Valdoxan. (2018 (2021). Valdoxan. https://www.ema.europa.eu/en/medicines/human/EPAR/valdoxan.

[6] Prescrire international (2013). Agomelatine: a review of adverse effects. Prescrire Int.

